# A Coordinated Research Agenda for Nature-Based Learning

**DOI:** 10.3389/fpsyg.2019.00766

**Published:** 2019-04-22

**Authors:** Cathy Jordan, Louise Chawla

**Affiliations:** ^1^Pediatrics and Extension, University of Minnesota, Minneapolis, MN, United States; ^2^Children & Nature Network, Minneapolis, MN, United States; ^3^Environmental Design, University of Colorado Boulder, Boulder, CO, United States

**Keywords:** nature-based learning, research agenda, children, academic outcomes, personal development, environmental stewardship

## Abstract

Evidence is mounting that nature-based learning (NBL) enhances children’s educational and developmental outcomes, making this an opportune time to identify promising questions to carry research and practice in this field forward. We present the outcomes of a process to set a research agenda for NBL, undertaken by the Science of Nature-Based Learning Collaborative Research Network, with funding from the National Science Foundation. A literature review and several approaches to gathering input from researchers, practitioners, and funders resulted in recommendations for research questions and methodological improvements to increase the relevance and rigor of research in this field. Some questions seek to understand how learning in nature affects what children learn, how they learn, and how it varies based on age, gender, socioeconomic status, ethnic background, special needs, and individual differences. Outcomes of interest cover academic performance, practical skills, personal development, and environmental stewardship. Other questions seek to find causal explanations for observed outcomes. To create optimal conditions for NBL, the research agenda includes practical questions about how to prepare teachers to work successfully in nature and how to support their adoption of this approach. Not least, the research agenda asks whether learning in nature can address major societal issues by moderating the effect of socioeconomic disadvantage on children’s academic achievement, personal development and wellbeing, and how these benefits might be attained at reasonable costs. A deeper understanding of how, why and for whom different forms of nature contact enhance learning and development is needed to guide practice and policy decision-making.

## Introduction

Although evidence is accumulating for the impact of nature-based learning (NBL) on children’s outcomes, there is much we don’t know (Kuo et al., 2019). A deeper understanding of how, why, for whom, and under what circumstances different forms of nature contact enhance learning and development is needed to guide practice and policy decision-making. This article presents the outcome of an initiative to define NBL and set a research agenda to advance the pace and rigor of research on its impact.

In 2015, the United States National Science Foundation (NSF) provided a 3-year grant to the University of Minnesota, the Children & Nature Network (C&NN), the North American Association for Environmental Education (NAAEE), and the University of Illinois Urbana-Champaign to establish the Science of Nature-Based Learning Collaborative Research Network (NBLR Network). On three occasions, the NBLR Network convened two dozen academic researchers from diverse disciplines, practitioners, environmental organization representatives, and funders from across the United States. The Network aimed to: (1) jointly develop a definition and research agenda to inform the rigorous development of the science of NBL, (2) disseminate research-based information, and (3) conduct collaborative research responsive to this agenda (Jordan et al., 2017). This article reports on the first aim of developing a definition and research agenda. It draws on an integrative literature review to determine and disseminate the status of our understanding of NBL impacts and explanatory mechanisms (see Kuo et al., 2019). Collaborative research that is responsive to agenda questions is currently underway.

The term “nature-based learning” was introduced in the grant application to NSF as part of an effort to coordinate research that had been scattered across multiple disciplines. NBLR Network members were sent a draft definition of this term by this article’s authors, and they responded with suggestions and comments. Successive revisions were circulated until members of the network agreed on the following definition and scope for this field.

Nature-based learning, or learning through exposure to nature and nature-based activities, occurs in natural settings and where elements of nature have been brought into built environments, such as plants, animals, and water. It encompasses the acquisition of knowledge, skills, values, attitudes, and behaviors in realms including, but not limited to, academic achievement, personal development, and environmental stewardship. It includes learning about the natural world, but extends to engagement in any subject, skill or interest while in natural surroundings. NBL can occur with varying degrees of guidance or structure, across the age span, alone or with others, and in urban, suburban, rural, and wilderness settings. NBL occurs in informal, non-formal, and formal settings (La Belle, 1982).^1^ With respect to children’s NBL, it includes *informal* learning during children’s free play or discovery in nature in their yards, near their homes, in green schoolyards, on the naturalized grounds of child care centers, or in any other natural area. It includes *non-formal* learning in nature during out-of-school programs, camps or family visits to parks or nature centers. And it includes *formal* learning when children have contact with nature during structured activities in schools, preschools, and child care centers, or during outdoor field trips.

The following section of this article reviews the methods used to develop an NBL research agenda. A subsequent section summarizes the agenda’s major questions grounded in the literature and in the minds of educators, researchers, and funders, as well as recommendations for methods, measures, and designs that will be complementary and rigorous. The intent of this article is to encourage more coordination and collaboration among researchers, to promote a focus on the most pertinent research questions and most robust methods in order to advance this field, and to make a case for the importance of NBL as a field for study as well as practice. We acknowledge the boundary that participants in this agenda-setting process were drawn from the United States. They considered existing studies from around the world and intended their work to be useful internationally; yet different countries may have different research cultures, and this agenda might reflect different emphases if it were generated in another part of the world.

## Methodology in Developing the Research Agenda

### Assembling Diverse Perspectives on NBL

This section traces the process of setting a research agenda during the 3-year period of the National Science Foundation grant that began in September 2015. The project’s coordinating team from the grant’s four lead institutions worked together to identify academic researchers, practitioners, representatives of environmental organizations, and funders from across the United States whose work related to NBL, with the goal of assembling a diverse membership for the NBLR Network, based on a variety of disciplines, methodological approaches, and stakeholder connections. The 23 members of the network first convened in November 2015 for a 3-day retreat to build relationships, agree on a common vision and direction for work, and discuss possibilities for interdisciplinary collaboration. In January 2016, NBLR Network members were asked to share written answers to the following questions, which guided development of the research agenda.

(1) What is the status of our knowledge about whether, how, why, under what circumstances and for whom nature impacts children’s learning?
(2) What are the strengths and limitations of the research?
(3) What research questions would most effectively advance knowledge relevant to practice and policy?
(4) Are there considerations about the state of the current research that suggest methodological recommendations for the field?

After members shared their written reflections, they participated in conference calls to further elaborate and interpret responses.

Several means were used to capture the ideas of funders and practitioners, beyond representatives of these groups in the NBLR Network. The May 2016 C&NN conference provided two opportunities for group discussion—the Blue Sky Funders’ Forum and an open forum for conference attendees. Both provided occasions to tap non-NBLR Network thinking regarding needs for additional research. The Natural Start Alliance nature-based preschool conference in August of 2016 and the Research Symposium associated with the October 2016 NAAEE conference offered opportunities for small group discussions with other constituencies regarding research gaps and needs. Finally, a member survey administered by NAAEE highlighted the work of the NBLR Network and collected additional input. For more details about NBLR Network strategies, processes for identifying and convening network members, members’ disciplines and fields of practice, and processes to gather information from other groups, see section “Network Participants and Processes” and Jordan et al. (2017).

### Network Participants and Processes

In September 2015, at the beginning of the National Science Foundation grant supporting the Science of Nature-Based Learning Collaborative Research Network (NBLR Network), the project’s coordinating team worked together to assemble diverse perspectives on research to understand and apply NBL. They invited researchers to the NBLR Network whose disciplines included educational science, cognitive science, early childhood education, environmental education, developmental psychology, ecopsychology, environmental psychology, environmental neuroscience, stress neurobiology, environmental design and landscape architecture. Researchers brought expertise in qualitative and quantitative methods, field observation studies, intervention studies, neuropsychological assessment, behavioral mapping, and participatory action research. Participants from other sectors included teachers, teacher educators, leaders of professional societies, funders, and science communicators. In addition to 18 invited individuals, the NBLR Network included Principal Investigators from the lead organizations and staff from C&NN, for a total of 23 members.

Network members gathered in person on three occasions: two working retreats in November 2015 and November 2016, and as part of an expanded NBL Research Action Area that was part of the C&NN Network Leadership Summit in June 2018. Outside of meetings, members communicated through email and regularly scheduled conference calls. Agenda setting was a major focus of the November 2016 retreat and communication during 2017 and early 2018.

During the May 2016 C&NN Conference, about 40 people participated in a Funders’ Forum that included representatives of organizations that might consider funding NBL research and application, along with a few members of the NBLR Network. After listening to a panel of speakers present existing evidence relevant to NBL and new areas for investigation, people broke into small groups for facilitated discussions. Discussions were guided by the questions: “What do you, as funders, feel you need to know to help make connecting kids to nature a higher priority for funding? Given the knowledge we already have, how could you support taking action to apply existing knowledge?” At each table, people began by writing down their individual responses to these questions, and then engaged in group discussions. Later in the forum, facilitators synthesized and reported back on key ideas that emerged from people’s written responses and discussions. This synthesis was shared with members of the NBLR Network.

At the same conference, several members of the NBLR Network convened a “research action lab” open to anyone attending the conference. About 100 people came, including educators, staff in nature-based non-profits, advocates for children and nature, researchers, and policy makers. They divided into five groups depending on the region where they worked in the United States or their international affiliation, and discussed the following questions: “What information do you need to make your efforts to connect children to nature to enhance learning outcomes more effective? How would you use that information? In what format do you want to receive the information resulting from the NBL Network’s efforts to set a research agenda for the field?” Note takers recorded participants’ responses and this information was reported back to the NBLR Network.

The August 2016 Natural Start Alliance conference for educators, school directors, and advocates for nature-based preschools, followed by the Research Symposium connected with the October 2016 NAAEE conference, offered opportunities for small group discussions about information that participants would find most helpful and important questions for NBL research. These discussions confirmed the value of questions that had already been identified by the preceding larger groups.

In the fall of 2016, NAAEE sent a survey to its members that included the questions: “How do you learn about new information in environmental education?” and “What kinds of information or research would help you develop, deliver, or refine programs to connect children to nature?” A group of NAAEE researchers created, pilot tested, and distributed the survey through naaee.org, eePRO (NAAEE’s online platform for environmental education professional development), mailings to NAAEE members and subscribers, and social media. The survey remained open for 3 weeks and two reminder mailings were sent during that time frame. A total of 167 respondents completed the survey. A summary of the findings was shared with the NBLR Network.

### Generating a Literature Review to Guide Agenda Discussions

During the summer of 2016, three members of the network prepared a research review of nature’s impact on academic functioning, personal development and environmental stewardship, as well as explanatory variables related to learners and learning contexts. This review of existing research was a necessary foundation for identifying promising directions for future research. Details about the review scope, scale and procedures, including search keywords and operational definitions of key terms, are provided in the review article by Kuo et al. (2019). The literature review consisted of three main phases, which are described here.

#### Phase 1

The first step was to utilize recent peer-reviewed research summaries relevant to NBL and identify major themes related to NBL at the time of their publication. Articles covered in these previous reviews were added to the review database. The purpose of this phase was to understand the previous state of the literature and the main themes in the literature at the time of past reviews’ publication.

#### Phase 2

The second step was to collect peer reviewed journal articles that were published since the cut-off dates for previous reviews. This research was limited to articles published in English, although the research may have been conducted anywhere in the world, and it included work that addressed any aspect of learning and developmental outcomes associated with any aspect of nature, utilizing a variety of research methods. At this time, the purpose was to update and expand findings from the previous review papers, and to present the diversity of the literature as a whole.

#### Phase 3

The third and last step to identify relevant research was intended to extend and deepen results of the preceding steps. It included two processes. Because some topic areas yielded only a few articles during the initial searches, specific searches were conducted to determine if these were in fact little studied areas or under-sampled by the preceding searches. Additionally, foundational papers and reviews were sought that shed light on potential mechanisms that connect nature and learning, though these publications may have come from general research on topics such as learning, cognitive science, or developmental outcomes. For example, if existing studies indicated that learning in nature sparked children’s curiosity, then there was a search for papers which reviewed the general role of curiosity in learning. The purpose was to create a cohesive narrative that suggested mechanisms through which nature might affect learning outcomes.

A link to a spreadsheet of the articles retrieved during these three phases of the literature review is reproduced here: https://goo.gl/FZ1CA9, as well as in the review by Kuo et al. (2019).

### Identifying Directions for Future Research

A draft of the literature review was presented at the second NBLR Network retreat in November 2016. Network members considered the review, along with results of their own written reflections and the input gathered through C&NN and NAAEE. People worked in small groups to develop focal areas and questions for the research agenda. Because their goal was to advance research that can be translated into educational policy and practice, members proposed the following criteria, in addition to feasibility, as they deliberated.

Research agenda questions should do one or more of the following:

(1) Address major social issues in a compelling way
(2) Affect large populations
(3) Cross developmental stages
(4) Translate into educational policy to help teachers and school administrators enhance students’ academic success
(5) Suggest how institutions can promote stewardship values and behaviors
(6) Help designers and urban planners create places where children can connect with nature in meaningful ways
(7) Achieve valued public goals in cost-effective ways, in some cases even saving public money

Applying these criteria, retreat attendees voted for questions they considered most important to advance the field of NBL.

During 2017, a report on the voting results and associated discussions was distributed to network members. Drawing on this report, reports on the C&NN conference Funders’ Forum and open forum, and NAAEE survey, the authors of this article condensed and categorized the questions generated, along with methodological recommendations, and circulated them to the NBLR Network in early 2018. Feedback was gathered through email and conference calls. Questions and recommendations developed as a result of this process, vetted by NBLR Network members, are presented in the sections below.

### Agenda Consensus and Challenges

Through NBLR Network discussions and input from the Funders’ Forum, 2016 C&NN Conference, and NAAEE survey, more questions were generated than a research agenda could accommodate, given its goal of bringing people together in coordinated, collaborative research rather than dispersing research efforts in many unconnected directions. The challenge of gaining consensus around a few key questions was addressed in several ways. At their November 2016 retreat, members of the NBLR Network began their review of research gaps and promising research directions by generating criteria for the most productive questions. They used these criteria as they reviewed the questions that they initially suggested in individual written reflections, as well as questions proposed by the funders, C&NN conference attendees, and NAAEE survey respondents. On this basis, they drafted questions that they posted on a wall and voted on. A report was generated that contained the resulting questions and summaries of associated discussions.

The authors of this article then took this report and the reports from other groups’ meetings—keeping agreed-on criteria in mind—and drafted Tables 1, 2 for this article. They sought to balance questions generated from the perspectives of research and practice, as the NBLR Network agreed on the importance of both sides. Reflecting contributors’ diverse backgrounds, questions that invited quantitative, qualitative, action research and mixed-methods were valued equally. Questions that were raised repeatedly were included; but to be consistent with the criteria agreed to by the NBLR Network, a focused effort was made to include questions relevant to the goals of promoting healthy child development, addressing important social and environmental issues, and guiding policy and practice. When the initial draft of Table 1 was circulated, some network members suggested that emphasis be given to questions with significant policy and practice applications by creating a second table. For this purpose, questions of this kind that network members highlighted were repeated or reworded in Table 2. Drafts of both tables were shared with network members, who discussed them via conference calls and email. The tables were revised and shared with the network again for final approval before inclusion in this article.

**Table 1:** A framework for research to advance the understanding and implementation of nature-based learning (NBL).

**A. Learning outcomes and differential effects**
**Learning outcomes**
How effectively do children learn content and skills through NBL compared with instruction in classrooms where nature is absent? • How do schools or classrooms that practice NBL compare with schools or classrooms without nature with respect to academic achievement, graduation rates, and student and parental satisfaction? • How do nature-based preschools and kindergartens compare with conventional early childhood programs that emphasize indoor learning in terms of preparing children for school readiness? • Are there situations when NBL is more effective and when classroom-based instruction is more effective? • How might NBL and classroom-based instruction complement each other? What is the range of learning outcomes influenced by nature? *Motivation to learn/knowledge gain/skill development/creativity/curiosity/cognitive processes such as attention, encoding, retention, recall/executive skills such as behavior regulation/social and emotional learning/reduced stress, improved mood and mental health/physical health/academic performance such as test scores and graduation rates/environmental stewardship values and behaviors^∗^* Does NBL contribute to stewardship values or conservation behaviors? Differential effects based on age, population group, and individual differences
**Learning outcomes**
How do age and developmental stage influence the relationship between nature and learning? • What are key elements of nature experiences important at different ages? • What different forms of knowledge, skills, values, attitudes, and behaviors develop in nature at different ages? • Are there critical windows for the development of different outcomes in nature? To promote academic achievement, personal development and environmental stewardship, what types of nature experiences are most appropriate at different ages? How does NBL affect special populations in terms of learning outcomes? • How does NBL affect children from socioeconomically disadvantaged families? • Does the impact of NBL differ based on historic relationships with nature grounded in cultural or ethnic background? • Are there gender differences in nature’s impact on children? • How does nature exposure impact learning for children with special needs such as ADHD, autism or learning disabilities? Are there individual differences in response to NBL? What determines why there may be different outcomes for children involved in the same experience?
**B. Mechanisms of influence**
What are the mechanisms that underlie the relationship between nature and learning? *More focused attention/improved behavior regulation/increased creativity/reduced stress/greater enthusiasm for and engagement in learning/increased physical activity/improved health and wellbeing/calmer, quieter learning context/more cooperative social context/opportunities for autonomous discovery and action/self-perception/self-identity/connection between content and the child’s locality/enhanced sense of purpose*^∗^ • What mediator variables explain the relationship between nature and learning outcomes, and what is the influence of different variables separately and in combination? • Is it possible to establish that nature impacts learning and development in a causal manner? • What moderator variables influence the strength of the relationship between nature and learning outcomes? Do mechanisms vary for different groups, in different contexts? If NBL has such differential effects, why? What are key elements of nature experiences that affect children? *Type of natural features/type of activities such as unstructured play and exploration, guided inquiry and adult-led instruction/degree of manipulation of natural elements/duration/frequency/individual or group experience/type of people with the child, such as teacher, parent, naturalist, classmates, friends/degree of teacher preparation and confidence in NBL approaches*^∗^ Does nature bring associated ingredients of learning together in a distinctive way? For example, does it bring opportunities for unstructured exploration, freedom to manipulate natural materials, creativity, and social cooperation together in a unique or synergistic way? How do interpersonal dynamics among children, parents, friends, and teachers influence NBL? How might power hierarchies or social stereotypes based on race, ethnicity, culture, class, gender or age influence NBL? What does nature do to the brain? • What are the channels of nature’s effects? *Sight/sound/smell/touch/emotion/movement*^∗^ • Does the impact of nature on the brain differ based on age? • Does nature contact influence the development of the brain in terms of structure or physiology?
What is the impact on learning when access to nature is reduced? *Removing recess in spaces with nature/no green views from school windows/more screen time*^∗^
**C. Implications for policy and practice**
Policy or practice What nature-based experiences are most appropriate for different developmental stages of childhood to achieve optimal learning outcomes? Can NBL play a role in reducing the opportunity gap and achievement gap between children from more and less advantaged backgrounds? How does nature compare with other programs and approaches that compete for educational funding in terms of its effectiveness in enhancing learning? What are the effects on learning of the cheapest and easiest ways of bringing nature into schools and day care centers? What are NBL best practices in different educational contexts? What evidence, messages, and strategies encourage increased demand for NBL and the application of NBL practices by educators, parents and other people who have influence over opportunities for children? What determines differences in access to nature, green school grounds, and NBL? Is NBL a social justice issue?
Preparation and professional development What are the best strategies for teachers to use to enhance student learning in nature? What are effective practices for preparing and supporting teachers and administrators in the adoption of NBL in their classrooms and schools? What are barriers to teachers’ and administrators’ adoption?
Technology augmented learning How does technology augment, simulate or mediate NBL? Are there costs as well as benefits? How does nature mediated or augmented through technology impact learning compared to experiences of real nature? Under what conditions is technology effective in enhancing nature’s impact on learning? How can we leverage technology to present nature in new ways for learning? How would new technologies function that do not substitute for nature, or for interaction with nature, but add additional forms of interaction?


*^∗^This list is suggestive, based on current evidence, but not necessarily complete*.

**Table 2:** Examples of “game-changing” research questions and justifications.

Question	Justification
Can nature reduce educational opportunity gaps and achievement gaps between children from different economic backgrounds?	Contact with nature shows an array of benefits for children across socioeconomic lines, at the same time as research shows that low-income families are more likely to live in urban neighborhoods with low levels of vegetation and smaller, less safe and less maintained parks, compared to middle- and high-income families (Jesdale et al., 2013; Chawla, 2015; Rigolon, 2017). Therefore, benefits of bringing children from disadvantaged backgrounds to nature and nature to their schools, child care centers and neighborhoods merits particular attention.
If learning in nature can enhance children’s achievement and wellbeing, how do its costs compare with other approaches that compete for educational funding?	Research is needed that analyzes the economic costs of NBL practices relative to other interventions that lack natural elements. Cost accounting should include the full valuation of NBL in terms of impact on academic achievement, physical health, mental health, behavioral function, engagement in learning, use of special education services, and interaction with the criminal justice system. A compelling case for NBL can be made if educational outcomes are similar to conventional approaches but produce cost-savings in additional arenas, and an even more compelling case if NBL can narrow gaps in educational outcomes compared to conventional approaches.
What are the mechanisms that underlie the relationship between nature and learning?	Understanding how contact with nature facilitates and improves learning will permit the effective and efficient delivery of NBL experiences and the design of natural areas to best promote learning and development. For example, if research shows that nature enhances learning by reducing stress, then programs and settings should be designed to activate this pathway: and similarly with other potential pathways such as more focused attention or more cooperative and supportive social dynamics.
How does nature impact the learning of children with special needs as a result of physical health, mental health, or cognitive conditions; learning differences; or educational disadvantages due to low income?	When individuals with special needs or disadvantages in the educational setting do not benefit from education as much as they could or do not find meaningful roles in society, there are high costs to those individuals, their families, school districts, and society in terms of expenses, lost potential and reduced well-being.
What teacher characteristics and practices enhance the association between NBL approaches and educational outcomes? How can teachers be prepared and supported to adopt NBL practices?	The impact of NBL is partially dependent on the attitudes, skills and practices of teachers (Mcfarland et al., 2013). Understanding how teachers learn to value NBL, integrate it into their school day, and promote positive outcomes will facilitate effective teacher preparation and professional development programs. This information will suggest how programs of teacher education and school administrators can best support the adoption and effective implementation of NBL strategies, in both pre-service and in-service settings.
What knowledge and experiences promote people’s motivation and competence to protect the integrity of natural landscapes and ecosystems? How can these experiences be integrated into NBL practices?	Information is gathering on many sides that basic systems of the biosphere that support human health and wellbeing and the survival of other species are rapidly deteriorating (Millenium Ecosystem Assessment, 2005; Intergovernmental Panel on Climate Change, 2014). An essential dimension of NBL is learning to understand and care for the natural world.
How can technology be most effectively harnessed to enhance the outcomes of NBL?	Technology is a common feature in current and future-looking educational programs; yet technology can be overused, resulting in reduced engagement in active, enriching activities (Singer et al., 2009), including those in nature and disrupting cognitive functioning and optimal mental health (Chassiakos et al., 2016). Therefore, it is important to understand how technology can be used as a tool to enhance nature experiences or to present nature while mitigating risks of overuse.


A limitation of this process was that there were no opportunities to reconvene participants in the Funders’ Forum, C&NN action lab, or NAAEE survey for their reviews of the draft tables. An inherent limitation is that the tables reflect the ideas of the people involved, whereas different collections of participants may have generated different results. Although contributors to the agenda setting process were composed of researchers who represented diverse disciplines, funders of child-nature related research and programming, and practitioners who provided children with nature experiences both in-school and out of school, an even more diverse group in terms of knowledge, expertise, interests, and cultural backgrounds may have provided additional perspectives on research directions. No members of the NBLR Network, for example, represented child psychiatry, social work or anthropology, and there may have been other relevant fields to consider. The network was limited to people with publications related to NBL, who were willing to commit to the considerable amount of time that network activities required, and by the funding available to bring people together. On the practical side, given the goal of creating a network of people who could hold productive whole-group and small-group discussions in person or via conference calls and email, it was necessary to contain the group to a number that enabled people to function in this way.

## Priority Research Questions

Table 1 presents the key research areas and questions that emerged through this agenda setting process, with three areas of emphasis: Learning Outcomes and Differential Effects, Mechanisms of Influence, and Implications for Policy and Practice. Where some contributors to the agenda approached a general question from specific perspectives, these variations on the general question are bulleted. Topics that suggest the range of areas that a question might explore are indicated in italics.

As authors of this paper, we have observed that the study of NBL reflects the convergence of two research traditions: one interested in the influence of experiences in nature on learning across the curriculum, personal development, and environmental stewardship; and the other concerned with the influence of natural settings and surroundings on conditions for learning. The first tradition has a long history. Fieldwork in nature to learn subjects like biology and geology is well established in environmental education and science education, and the resurgence of school ground greening and school gardens has created conditions for “fieldwork” immediately outside school doors (for research reviews of different forms of outdoor learning, see Dillon et al., 2006; Williams and Dixon, 2013; Stern et al., 2014; Malone and Waite, 2016; Becker et al., 2017). The use of the environment as an integrating context to engage students in math, science, social studies, language arts and other disciplines as they study the world beyond school walls, including natural areas, is the domain of place-based education (Smith and Sobel, 2010; Chawla and Derr, 2012). There is also a long history of observations of children’s informal learning as they play and explore on natural school grounds and find nature in their local environment (Chawla, 2015). The questions in Table 1 indicate that many aspects of outdoor learning still need to be better understood, but work in this area has much to build on as it moves forward.

The second tradition—investigating the influence of nature on conditions for learning—has emerged recently, demonstrating that vegetation and other elements of nature in classrooms, on school grounds, and in the proximity of schools are associated with more effective cognitive functioning, decreased stress, improved health, and enhanced classroom and social learning environments—all of which can facilitate learning and higher student achievement (see reviews by Chawla, 2015; Gifford and Chen, 2016; Becker et al., 2017; Kuo et al., 2019). Many studies of this topic suggest productive directions for further investigation. Whereas the first research tradition focuses on learning in nature to enhance knowledge, skills and personal development, this second tradition involves children’s basic wellbeing and capacity to learn efficiently. Recently, and partly with the assistance of the NSF grant to promote the Science of Nature-Based Learning, people from these different backgrounds have been sharing their work at conferences and other professional meetings.

The questions in Table 1 suggest an ambitious agenda for moving an understanding of NBL forward. They seek to understand how learning in nature affects what children learn, how they learn, and how it varies based on age, gender, socioeconomic status, ethnic background, special needs, and individual differences. They investigate the relative benefits of learning in nature and through conventional classroom-based instruction, and learning in settings where there is nature in and around buildings with learning in predominantly hardscaped, built surroundings. Outcomes of interest cover academic performance, practical skills, personal development, and environmental stewardship. Other questions seek to identify mechanisms of action in NBL and find causal explanations for the outcomes observed. To create effective conditions for NBL, the research agenda includes a number of practical questions about how to prepare teachers to work successfully in nature and encourage their adoption of this approach. Possibilities for using technology to augment learning in nature also merit exploration (such as approaches identified in Kahn, 2011). Not least, the research agenda asks whether learning in nature can address major societal issues by moderating the effect of socioeconomic disadvantage on children’s outcomes, and how these benefits might be attained at reasonable costs. Although these questions outline an ambitious agenda for future research, promising results of past studies suggest that further investment in this field may significantly benefit children and their societies.

In drafting this research agenda, funders, researchers who focus on school-based initiatives, and practitioners emphasized the importance of systematically investigating how to most effectively disseminate results of NBL research and encourage implementation. It is important to match growing evidence of benefits of learning in nature with outreach to teachers, school administrators, school boards, schools of education, child care center directors and people in other institutions who have opportunities to apply nature-based approaches. Effective outreach depends on understanding barriers to the integration of NBL into teacher preparation and practice, how barriers can be lowered, and the types of data and messages that will help practitioners understand the value of NBL. Similar questions need to be asked relative to reaching the public at large, in order to build public support for NBL.

Though not comprehensive, the questions offered in the research agenda have the potential to significantly advance our knowledge and ability to inform policy and practice in an array of areas. Given the wide range of subjects covered by the questions proposed for this research agenda, it is reasonable to ask where to begin or what to prioritize. In Table 2, we offer a set of “game-changing” questions—research questions that are most likely to yield critical information for practice and policy decision-making.

## Recommendations for Future Research Approaches

Significant scientific advances are made not only by asking the most relevant and important questions, but by utilizing approaches that will yield the most useful, valid and reliable information. What general recommendations can be made to strengthen future research in this field?

The researchers, practitioners, and funders who helped define this research agenda recommend a more coordinated approach to NBL research in the future. In part, this will require periodic syntheses of what is already known in relation to the questions in Tables 1, 2, to guide further efforts to fill in gaps in understanding. To facilitate research syntheses, C&NN established an online Research Library that deposits, on an ongoing basis, lay summaries of new studies related to NBL as well as other aspects of children’s relationship with nature^2^. C&NN’s monthly Research Digest has begun to curate existing research on selected themes, such as equitable access to nature’s benefits^3^. C&NN and NAAEE now provide a central location to access the combined resources of C&NN’s and NAAEE’s research libraries^4^ to provide comprehensive coverage of the two traditions of investigation reflected in this research agenda.

More coordinated research will also require the consistent use of adequate descriptions of study contexts as well as consistent measures of study variables (see also Kuo et al., 2019). Qualitative and quantitative researchers need to specify learning settings and activities, including elements of nature in each setting, length of children’s time in nature, and how children engage with nature—whether it is a passive view or background, or they use it actively through their own autonomous exploration or encounters facilitated by teachers, peers or other people. Complete descriptions are important for understanding and applying results and identifying potential causal mechanisms that underlie learning.

Coordinated progress in quantitative research and experimental designs will be furthered by agreement on valid, reliable measures of nature exposure, mediating variables and learning outcomes. Many measures already exist, and they need to be evaluated to understand which are most effective with different age groups and in different learning contexts. A working group is underway to do this for measures of nature connection, but similar evaluations are needed of other key variables important for this research agenda. It would be helpful to have an online bank of NBL measures that researchers can draw from, along with examples of studies where they have been applied and recommendations for their appropriate use. This would encourage more reliable comparisons across studies.

Nature-based learning research needs to move forward through complementary methodological approaches. Different methods are required to investigate questions of different kinds, and therefore the field of NBL will be advanced most effectively by different methods and mixed-method approaches. For example, to understand how NBL and classroom-based approaches compare or complement each other, it can be helpful to begin with observations and interviews with teachers and students, in order to identify similarities and differences. Qualitative results may suggest how settings with and without nature afford different opportunities for teaching and learning, which may lead to different outcomes; and these outcomes can then be tested in more controlled ways through experimental designs. Experimental designs can also investigate the mechanisms that underlie results. As experiments and correlational studies establish with increasing confidence key variables that affect learning, the case builds for investments in longitudinal research that can track the effect of key variables over time. Some objectives, such as quantifying the effect of learning in nature preschools on performance in elementary school, can be addressed with relatively short-term studies; others, such as tracing the effect of childhood learning in nature childhood learning on environmental stewardship values and behaviors in adulthood, require long-term studies.

Nature-based learning research will be advanced through collaboration between academic researchers and practitioners and through multidisciplinary and multiethnic perspectives. In participatory research, practitioners, parents and young people themselves can help at different stages of research, including defining questions, designing and implementing studies, interpreting results, and disseminating outcomes. The audiences that researchers seek to reach are best qualified to identify the type of information that will catch their attention and resonate with their values and practical considerations. For example, the experiment reported by Kuo et al. (2018) was designed to test the validity of teachers’ common fear that if they take a class to an outdoor setting in nature, students will never settle down to concentrate on lessons after they return to the school building (finding, in contrast, that students concentrated better in their subsequent indoor class). In a similar way, researchers can identify NBL outcomes that matter most to teachers, school administrators, parents and children themselves as promising directions for research efforts.

## Conclusion

Existing research suggests that NBL has many positive outcomes for children’s learning and development. It suggests promising directions for future investigation; but to move forward, NBL research will benefit from a clear definition and a coordinated agenda. This paper has attempted to provide this framework by presenting a definition and a list of priority questions that have been drafted and reviewed by academic researchers from diverse disciplines, practitioners, environmental organization representatives, and funders.

Priority questions for future research cluster into three domains: (1) learning outcomes, including understanding how learning in nature compares with learning in classrooms, preschools and child care centers, and how outcomes may vary by age, gender, socioeconomic background, ethnic background, individual differences, or special needs; (2) the mechanisms that explain relationships between nature and learning; and (3) how to most effectively apply research to policy and practice. This Research Agenda also suggests that a few questions have the potential of uncovering relationships between nature and learning that could have “game changing” effects on the practices of policy makers, educators, school administrators, urban planners, designers, staff in nature centers and parks, parents, and other people who influence children’s access to nature. With the aim of enhancing conditions for children’s learning and development, this agenda seeks to accelerate progress on the science of NBL.

## Author Contributions

CJ served as the principal investigator of the Science of Nature-Based Learning Collaborative Research Network project. She surveyed the NBLR Network members, conducted discussion sessions at conferences, and supervised the graduate student conducting the literature review. She was involved in the generation and review of research questions, co-wrote the manuscript, and solicited input from NBLR Network. LC was a member of the NBLR Network. She was involved in the generation and review of research questions and co-wrote the manuscript.

## Conflict of Interest Statement

The authors declare that the research was conducted in the absence of any commercial or financial relationships that could be construed as a potential conflict of interest.

## Footnotes

1 In the United States, the National Science Foundation distinguishes *formal* and *informal* learning, putting *non-formal* and *informal* in one category. The three-part distinction among *formal, non-formal*, and *informal*, used here, which is widely used in Europe and the work of UNESCO, better reflects the diversity of practices in the NBLR Network.

2 childrenandnature.org/research-library

3 https://www.childrenandnature.org/learn/research-digest/

4 naaee.org/eeresearch
